# Correction: Kobata et al. Situational Factors Impacting Harmful Behavior Towards Others Related to Mental Health in the Community and Their Associations: A Scoping Review Based on Systematic Reviews. *Healthcare* 2025, *13*, 152

**DOI:** 10.3390/healthcare13101129

**Published:** 2025-05-13

**Authors:** Issho Kobata, Yoshitomo Fukuura, Yuzaburo Kaba, Yukako Shigematsu

**Affiliations:** 1 Department of Nursing, Kurume University Graduate School of Medicine, 777-1 Higashikushiharamachi, Kurume-Shi 830-0003, Fukuoka, Japan; 2 Department of Nursing, School of Medicine, Kurume University, 777-1 Higashikushiharamachi, Kurume-Shi 830-0003, Fukuoka, Japan; fukuura_yoshitomo@kurume-u.ac.jp (Y.F.); kaba1208@med.kurume-u.ac.jp (Y.K.); shigematsu_yukako@med.kurume-u.ac.jp (Y.S.)

Due to clerical oversights during the manuscript preparation and editing process, there were some errors in the original publication [1], including inaccuracies in data reporting, incorrect citation placement, and typographical inconsistencies. 

## Text Correction

A correction has been made to the Abstract. The text “and forensic patients” has been removed:

**Abstract:** Background/Objectives: This study aimed to identify factors associated with harmful behavior toward others based on existing research. Methods: This scoping review focused on individuals at risk of harming others due to mental health issues, with the target population encompassing three settings: the community, inpatient facilities with frequent admissions and discharges, and healthcare settings where medical treatment is sought. A scoping review was conducted according to the Preferred Reporting Items for Systematic reviews and Meta-Analyses extension for Scoping Reviews. The terms violence, aggression, problem behavior, and workplace violence were used to search for related literature, subsequently selecting systematic reviews. Results: A total of 24 papers were ultimately included. From the included papers, background factors (demographic, personal history, and clinical aspects); situational factors (social connection status, daily life status); psychological factors; antecedents of harmful behavior; and triggers of harmful behavior were extracted as factors associated with harmful behavior. Conclusions: Our results indicate that background and situational factors lead to harmful behavior toward others, disruptions in the harmony between these factors cause disturbances in psychological processes, and harmful behavior toward others is triggered by stimuli that promote such behavior. Considering that all studies reviewed herein involved inpatients in medical settings, further research is required to identify the factors associated with harmful behaviors occurring in the community.

There were errors in the number of studies identified and excluded in the Results section. A correction has been made to the Results section, 3.1. Overview of the Included Studies, paragraph 1 (change 7073 to 7045, change 6907 to 6879):

The literature search identified 7045 papers. The literature for this scoping review consists of articles that examine relevant factors contributing to harmful behavior toward others in the community, particularly in situations where mental health welfare professionals are likely to be involved. Literature on child and elder abuse, literature on drug treatment and seclusion restraints, literature on staff-to-patient and staff-to-staff violence, and literature not related to community or mental illness were excluded, because they did not fit the theme and conceptual scope of this review. After excluding 6879 and 142 papers in the primary and secondary screening, respectively, 24 papers were ultimately included (Figure 1).

The text “and correctional facilities” has been removed from the Results section, 3.1. Overview of the Included Studies, paragraph 2:

Among the papers included herein, all of which were published between 2007 and 2023, 6 focused on general behaviors such as violence and aggression [3,8,12,13,18,19], 13 articles focused on behaviors among patients with psychiatric disorders [2,9,11,15,20–28], and 5 focused on rating scales for factors related to harmful behaviors toward others [1,29–32]. The scope of our study included papers involving inpatients, those focusing on the community, and those in medical settings.

A correction has been made to the Result section, 3.2. Factors Associated with Harmful Behavior Toward Others, paragraph 2. The text “<Demographic and environmental risk factors>” has been revised to “<Demographic and environmental factors>”. The text “[history of crime/criminal activity]” has been revised to “[history of criminal/criminal activity]”. The text “<family history (mental illness/substance use problem/crime)>” has been revised to “<family history (mental illness/substance use problems/criminal involvement)>”. The text “<intelligence and history of schooling>” has been revised to “<intelligence/history of school>”:

{Background factors (demographic and personal history)} were extracted from the following seven subcategories: <Demographic and environmental factors>, which includes four codes, such as [age]; <history of self-injurious/other harmful behavior>, which includes two codes, such as [history of violence]; <apprehension/criminal history>, which includes three codes, such as [history of criminal/criminal activity]; <violence victimization/experienced abuse>, which includes three codes, such as [experience of violence victimization]; <family history (mental illness/substance use problems/criminal involvement)>, which includes three codes, such as [family history of mental illness]; <intelligence/history of school>, which includes two codes, such as [intelligence]; and <secure attachment and conduct problems during childhood>, which includes three codes, such as [secure attachment in childhood/stable nurturing environment].

A correction has been made to the Conclusions section, paragraph 3. The text “and forensic patients” has been removed:

Considering that all the studies reviewed herein involved inpatients in medical settings, further research is required to identify the factors associated with harmful behaviors occurring in the community.

## Error in Figure

There was an error in Figure 1: PRISMA flowchart for the article selection process in the number of studies excluded (change from 6907 to 6879). The corrected version of Figure 1 is shown below. 

## Error in Citation

In the Results section, 3.1. Overview of the Included Studies, paragraph 2, the citation of reference 7 was wrongly added here. It should be reference 9:

Among the papers included herein, all of which were published between 2007 and 2023, 6 focused on general behaviors such as violence and aggression [3,8,12,13,18,19], 13 articles focused on behaviors among patients with psychiatric disorders [2,9,11,15,20–28], and 5 focused on rating scales for factors related to harmful behaviors toward others [1,29–32]. The scope of our study included papers involving inpatients, those focusing on the community, and those in medical settings.

The authors state that the scientific conclusions are unaffected. This correction was approved by the Academic Editor. The original publication has also been updated.

## Figures and Tables

**Figure 1:**
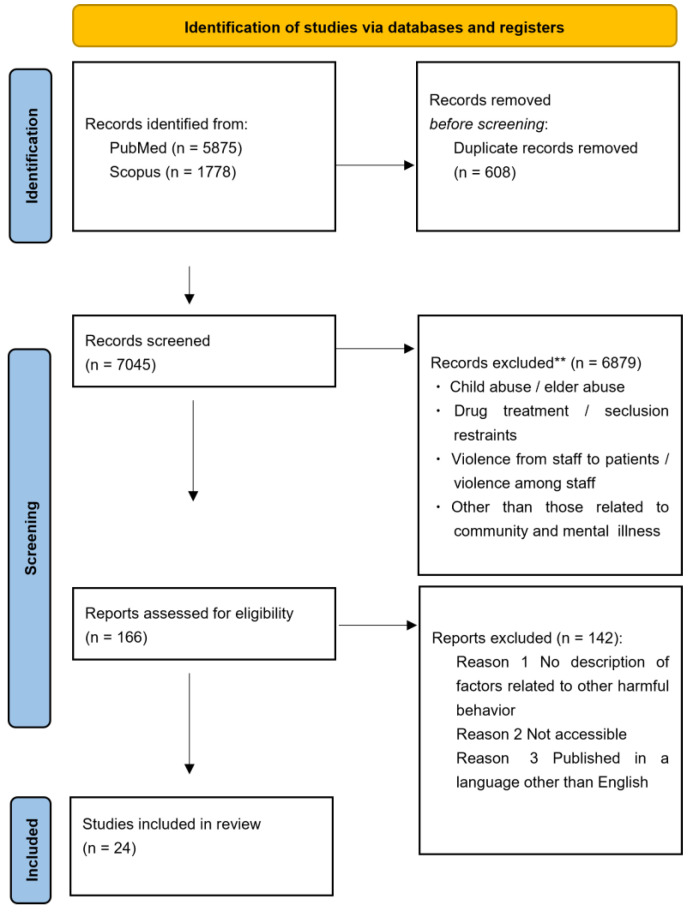
PRISMA flowchart for the article selection process. ** Based on title/abstract screening.
